# Study of Microstructure and Mechanical Properties of 800H Alloy During Creep

**DOI:** 10.3390/ma18040912

**Published:** 2025-02-19

**Authors:** Menglin Gao, Shengjun Xia, Chunfa Huang, Xing Hu, Shuaiheng Liang, Wenlu Zhang, Qiulin Li

**Affiliations:** 1 Institute of Materials Research, Shenzhen International Graduate School, Tsinghua University, Shenzhen 518055, China; 2 School of Materials Science and Engineering, Tsinghua University, Beijing 100084, China

**Keywords:** creep, microstructure evolution, Young’s modulus, quantitative model

## Abstract

Creep is one of the primary degradation mechanisms affecting the performance of the 800H alloy under long-term high-temperature stress conditions. Understanding the microstructural evolution during creep and developing a quantitative model to relate these changes to mechanical properties are essential for assessing creep damage and ensuring the safe operation of high-temperature equipment. By conducting a multiscale quantitative characterization of the microstructures in the 800H alloy across different creep stages, we systematically examined the evolution of various microstructural features and their influence on Young’s modulus. A quantitative prediction model of Young’s modulus based on microstructural characteristics was developed, achieving a prediction accuracy exceeding 95% with a mean absolute percentage error of just 1.59% compared to experimental values. This work not only elucidates the intrinsic relationship between microstructural features and macroscopic mechanical properties but also provides a foundation for the in-service creep damage assessment of high-temperature components.

## 1. Introduction

The 800H alloy, an austenitic Fe-Ni-Cr superalloy, is extensively utilized in nuclear power plants and other high-temperature industrial equipment due to its excellent high-temperature mechanical properties [1,2,3,4]. Prolonged exposure to high-temperature stress results in creep being the dominant degradation mechanism, and the microstructural evolution caused by creep significantly influences the mechanical properties of the alloy [5,6]. Among these properties, Young’s modulus is a key parameter for assessing material stiffness and evaluating service performance [7,8]. Furthermore, changes in Young’s modulus can be sensitively detected using nondestructive testing techniques, such as ultrasonic testing [9,10,11]. Consequently, investigating microstructural evolution and establishing a quantitative relationship between these features and Young’s modulus provide valuable insights and a robust foundation for assessing in-service creep damage.

In recent years, numerous studies have discussed the effects of various scales and types of microstructures—including grain size [12,13], different precipitates [14,15,16,17], dislocations [18,19], inclusions [20,21], and texture [22,23]—on Young’s modulus. However, most of these studies are qualitative, and quantitative studies are comparatively rare. Villuendas et al. [24] investigated the evolution of Young’s modulus after various plastic deformations in aluminum alloys, establishing a quantitative relationship between dislocation density, dislocation line length, and Young’s modulus during cold working. Xu et al. [25] explored the effect of precipitates on the Young’s modulus of magnesium alloys and developed a quantitative relationship between the precipitate fraction and Young’s modulus based on empirical and semi-empirical models. Although these studies successfully built quantitative models relating Young’s modulus to a single type of microstructure, the microstructural evolution of the 800H alloy during creep is far more complex, involving grain growth and the nucleation, growth, and dissolution of precipitates, as well as the formation and annihilation of dislocations [26,27,28,29]. Thus, analyzing the combined effects of various microstructures on the Young’s modulus and developing multiscale quantitative models remain pressing research challenges.

Here, we conducted interrupted creep tests on the 800H alloy to obtain samples from different creep stages and utilized multiple characterization techniques to elucidate the microstructural evolution and gather quantitative data. Additionally, nanoindentation was employed to precisely measure the changes in Young’s modulus across the creep stages. By integrating microstructural quantitative data, we systematically assessed the influence of various microstructures on Young’s modulus. Based on these insights, we developed a quantitative prediction model for Young’s modulus, achieving over 95% accuracy and demonstrating strong potential for high-temperature alloy performance prediction. This work not only offers a new perspective on understanding the creep behavior of the 800H alloy but also provides scientific support for using nondestructive testing to assess creep damage states.

## 2. Materials and Methods

**Figure 1:**
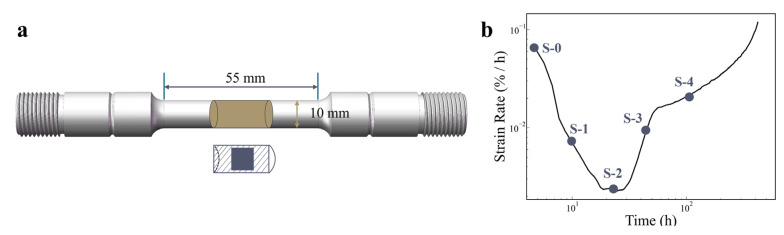
(**a**) Schematic of the creep sample dimensions and small square specimen extraction location, and (**b**) creep strain rate curve with sample points for interrupted creep tests.

The 800H alloy investigated in this study has a nominal chemical composition of Fe-31.8Ni-22.2Cr-0.09C-0.43Al-0.38Ti (wt.%). The material was solution-treated at 1130 °C for 10 h, followed by water quenching. Subsequently, the material was further machined into standard creep specimens with a gauge length of 55 mm and a gauge diameter of 10 mm, as illustrated in Figure 1a. Based on the experimental creep strain rate versus time curve obtained at 750 °C and 95 MPa, four interrupted creep test points—10, 25, 45, and 100 h (labeled S-1, S-2, S-3, and S-4, respectively)—were selected to represent different creep stages, whereas S-0 denotes the as-received sample (Figure 1b). All the creep tests were performed on a microcomputer-controlled mechanical creep testing machine. Small square specimens were cut from the longitudinal cross-section of the gauge area of each creep sample using a wire electrical discharge machine (Figure 1a). These samples were then mechanically polished with 400–1200-grit abrasive paper and subjected to electrolytic polishing at −20 °C under a constant voltage of 35 V for 40 s. The polishing electrolyte consisted of 10% perchloric acid in alcohol.

A scanning electron microscope (SEM, Apreo 2S, Thermo Fisher Scientific, Waltham, MA, USA) operated at 15 kV in backscattered electron mode was used to examine precipitates during the creep process. At least 20 images of random areas were captured per sample, and the precipitate volume (area) fraction was determined using ImageJ (Open source software, v.1.54a).

A high-resolution X-ray diffractometer (XRD, SmartLab, Rigaku, Tokyo, Japan) with Cu-Kα radiation was employed to measure the dislocation density. Diffraction patterns were acquired over a 2θ range of 40° to 120° at a scanning speed of 5°/min and a step size of 0.01°, focusing on the (111), (200), (220), (311), (222), and (400) crystallographic planes of austenite.

To obtain the Young’s modulus corresponding to the regions where microstructural characterization was performed, the nanoindentation technique was specifically selected.

A nanoindenter (G200, Keysight Technologies, Santa Rosa, CA, USA) was utilized to evaluate the Young’s modulus of the samples using a Berkovich tip and quasi-static indentation method. The maximum load applied was 10 gf, with a loading duration of 25 s and a peak hold time of 10 s. For each sample, 10 random points were tested within the same characterized area, and the average value was subsequently calculated.

For the transmission electron microscope (TEM, JEM3200FS, JEOL, Tokyo, Japan) analysis of dislocation configurations at various creep stages, 3 mm disks were punched from square samples, mechanically thinned to approximately 60 μm, and then further thinned using an electrolytic double-jet polisher under the following conditions: −20 °C, 16 V, and 10% perchloric acid in alcohol as the electrolyte. Bright-field images were taken at an accelerating voltage of 200 kV. For each sample, at least 20 images of random regions were collected, and ImageJ was used to measure the average length of dislocation.

## 3. Results and Discussion

### 3.1. Microstructure Characterization and Statistics

**Figure 2:**
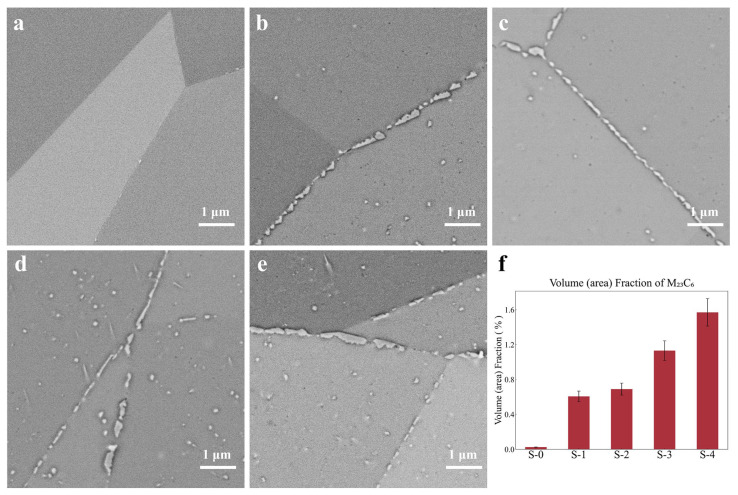
SEM characterization results of typical regions in various samples: (**a**) S-0, (**b**) S-1, (**c**) S-2, (**d**) S-3, and (**e**) S-4. (**f**) Volume (area) fraction of M_23_C_6_.

Figure 2a–e present SEM images of representative regions of samples at different creep stages. The white precipitates observed at grain boundaries and within grains are identified as M_23_C_6_, a Cr-rich precipitate with an FCC structure. This is one of the primary precipitates formed during the creep process of the 800H alloy, and its morphology and distribution are closely related to the creep resistance of the 800H alloy [30]. As shown in Figure 2a, the as-received sample exhibits nearly no M_23_C_6_ due to the prior solution treatment. In the early and intermediate creep stages (Figure 2b,c), M_23_C_6_ initially precipitates at the austenitic grain boundaries, forming chain-like structures that effectively pin the boundaries, thereby enhancing creep resistance and progressively reducing the creep rate. Only a small number of spherical M_23_C_6_ particles are distributed within the grains. In the late stages of creep (Figure 2d,e), M_23_C_6_ also begins to precipitate along some twin boundaries, while the chain-like M_23_C_6_ at austenitic grain boundaries starts to break and coarsen, indicating significant boundary sliding and an increased creep rate. Grain deformation causes some spherical M_23_C_6_ to transform into needle-like morphologies. Additionally, local stress concentration induced by deformation provides additional nucleation sites for M_23_C_6_ within the grains, substantially increasing intragranular M_23_C_6_ and reducing the ductility of the 800H alloy. Figure 2f illustrates the volume (area) fraction of M_23_C_6_ in each creep sample, based on a statistical analysis of 20 images. This quantitative information not only further validates the evolution pattern of the precipitates described above, but also serves as a foundational dataset for developing quantitative models linking microstructure to mechanical properties. Another precipitate, Ti(C,N), with a larger size (~0.5 μm to ~10 μm), is formed during solidification and shows no significant change during the creep process [31,32]. Therefore, it is not analyzed or discussed in this work.

**Figure 3:**
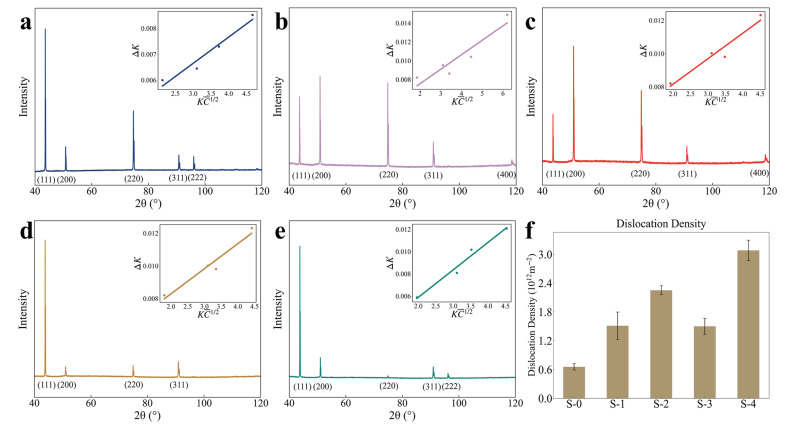
XRD patterns of various samples: (**a**) S-0, (**b**) S-1, (**c**) S-2, (**d**) S-3, and (**e**) S-4. (**f**) Calculated dislocation density.

The presence of dislocations influences the line broadening of X-ray diffraction peaks. Therefore, in this study, the dislocation density of samples at different creep stages was calculated from XRD patterns using the Modified Williamson–Hall (MWH) method. The MWH equation is expressed as [33]

(1) $$\Delta K=\alpha +\frac{\pi Mb^{2}}{2}\rho^{\frac{1}{2}}K{\bar{C}}^{\frac{1}{2}}+O\left(K^{2}\bar{C}\right)$$

where ρ is the dislocation density, K denotes the magnitude of the diffraction vector, calculated as K=2sinθ/λ, and θ and λ represent the diffraction angle and X-ray wavelength, respectively. ΔK can be expressed as ΔK=2cosθ(Δθ)/λ, and Δθ is the full width at half maximum (FWHM). The constants M and O are determined by the effective cut-off radius of dislocations [34]. For austenite, the Burgers vector b is 0.25 nm. The dislocation contrast factor ($\bar{C}$) for a given (hkl) diffraction peak is defined as

(2) $$\bar{C}={\bar{C}}_{h00}\left(1-qH^{2}\right)$$

(3) $$H^{2}=\frac{h^{2}k^{2}+k^{2}l^{2}+l^{2}h^{2}}{{\left(h^{2}+k^{2}+l^{2}\right)}^{2}}$$

where ${\bar{C}}_{h00}$ represents the average dislocation contrast factor for the (h00) diffraction, which is 0.301 in austenitic alloys [35,36], and q is a constant derived from fitting the XRD pattern data, which is subsequently used to determine the value of $KC^{1/2}$. According to Equation (1), ΔK can be regarded as a linear function of $KC^{1/2}$. By fitting data from at least four diffraction peaks, as shown in Figure 3a–e, the dislocation density ρ can be calculated using the slope β:

(4) $$\rho =\frac{2\beta }{\pi M^{2}b^{2}}$$

The standard error of the slope β is obtained from the covariance matrix during the fitting process. This standard error quantifies the uncertainty in β and is then propagated through Equation (4) to estimate the error in the dislocation density ρ.

Figure 3f depicts the dislocation density at various creep stages, showing a clear connection to precipitate evolution. In the early stages of creep (S-1, S-2), a rapid formation of precipitates, as previously described, effectively impedes dislocation motion and leads to a continuous rise in dislocation density. After reaching the peak, prolonged high temperatures and stress conditions promote climb-dominated mechanisms, causing some dislocations to begin to merge and be annihilated [37], which in turn lowers the dislocation density (S-3). Nevertheless, the ongoing accumulation of creep damage subsequently drives another increase in dislocation density (S-4).

**Figure 4:**
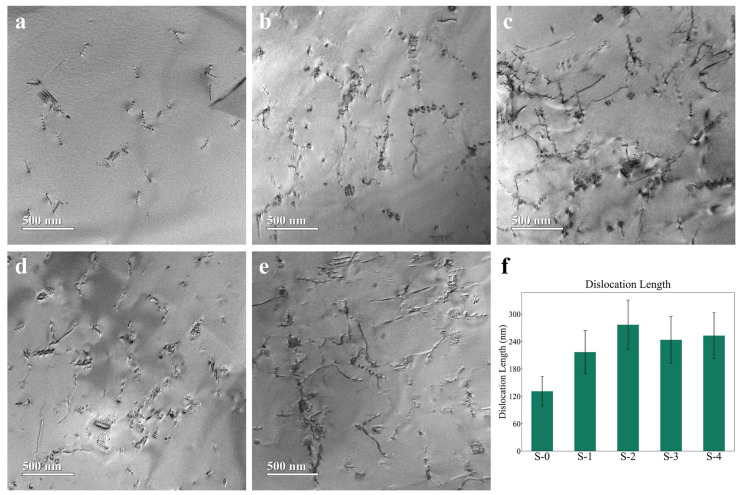
TEM characterization results of typical regions in various samples: (**a**) S-0, (**b**) S-1, (**c**) S-2, (**d**) S-3, and (**e**) S-4. (**f**) Dislocation length.

Figure 4a–e show TEM images of representative regions at different creep stages, revealing distinct dislocation configurations. In the as-received sample, only a few short dislocation lines are observed due to water quenching during the solution treatment process (Figure 4a). These dislocations serve as favorable nucleation sites for M_23_C_6_. During creep, the dislocation lines are elongated due to obstruction by these precipitates, while the Orowan bypass mechanism generates a few dislocation loops during the early creep stage (Figure 4b,c). As previously mentioned, when dislocation climb dominates, dislocation loops accommodate strain through flip–flop motion and are eventually annihilated [37]. Meanwhile, the length of dislocation lines decreases somewhat during this stage (Figure 4d,e). Through the use of ImageJ, the average length of dislocation lines for various creep stages can be obtained by analyzing 20 distinct regions for each sample, as shown in Figure 4f, laying a foundation for subsequent model development.

### 3.2. Mechanical Property Analysis and Modeling

**Figure 5:**
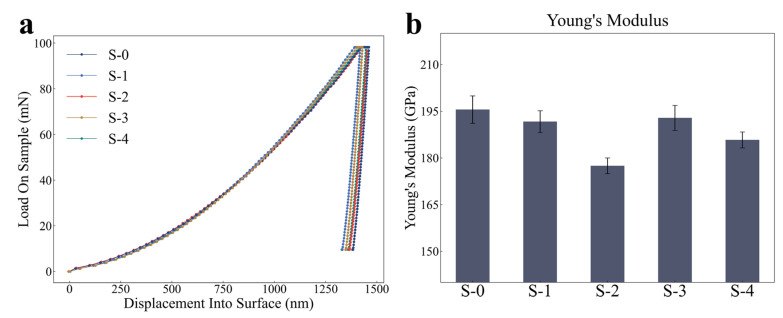
(**a**) Load–displacement curves of various samples and (**b**) extracted Young’s modulus of each sample.

Figure 5a presents the load–displacement curves of creep samples at various stages. The effective Young’s modulus, $E_{eff}$, representing the composite modulus of the indenter and sample, can be determined by the following equation [38]:

(5) $$E_{eff}=\frac{\sqrt{\pi }}{2}\frac{dP}{dh}\frac{1}{\sqrt{A}}$$

where P is the indentation load on samples, h is the displacement into the surface, and dP/dh represents the slope of the unloading segment of the load–displacement curve. A denotes the projected contact area between the indenter and the specimen. The relationship between the effective Young’s modulus and the modulus of the indenter and the sample can be expressed as [39]

(6) $$\frac{1}{E_{eff}}=\frac{1-v^{2}}{E}+\frac{1+v_{i}^{2}}{E_{i}}$$

where $E_{i}$ and $v_{i}$ represent the Young’s modulus and Poisson’s ratio of the indenter, which are 1140 GPa and 0.07, respectively, while v, the Poisson’s ratio of the sample, is equal to 0.3 in this work [40]. The Young’s modulus of samples, E, can be further extracted, as illustrated in Figure 5b.

During the creep process, the Young’s modulus exhibits a complex trend, with an initial decrease, followed by an increase, and then another decrease, which is closely related to microstructural evolution. Since grain size significantly affects Young’s modulus only when it is smaller than 20 nm [41], and the average grain size of the 800H alloy is much larger than 20 nm, this study disregards the effect of grain size on Young’s modulus. Instead, it focuses on the effects of precipitates and dislocations on Young’s modulus and develops a physics-based quantitative model.

The effect of dislocations mainly influences the Young’s modulus of the matrix. According to the relationship proposed by Mott [42] and Friedel [43], which correlates the dislocation density and dislocation line length with changes in Young’s modulus, the Young’s modulus of the sample matrix $E_{M}$ can be expressed as follows:

(7) $$E_{M}=E_{0}\left[1-\rho \left(\frac{l^{2}}{\alpha }\right)\right]$$

where $E_{0}$ denotes the matrix Young’s modulus under an ideal dislocation-free condition. For the 800H alloy, $E_{0}$ is set to 200 GPa [44]. ρ is the dislocation density, l is the length of the dislocation line, and α is an optimizable constant. The effect of precipitates on Young’s modulus is mainly attributed to the difference between the modulus of the precipitates and that of the matrix [25]. The Young’s modulus of the sample E, incorporating the influence of precipitates, can be determined using the widely applied Halpin–Tsai equation [45]:

(8) $$E=\frac{E_{M}\left(1+\beta \eta f_{p}\right)}{1-\eta f_{p}}$$

(9) $$\eta =\frac{E_{p}/E_{M}-1}{E_{p}/E_{M}+\beta }$$

where $f_{p}$ represents the fraction of precipitates, while $E_{p}$ refers to the Young’s modulus of the precipitate, specifically Cr_23_C_6_. According to the first-principles calculations by Sun and Li [46,47], $E_{p}$ is set to 375.1 GPa in this work. β is an optimizable constant. Substituting the matrix Young’s modulus $E_{M}$ calculated from Equation (7) into Equations (8) and (9), the final Young’s modulus of the sample can be derived.

**Figure 6:**
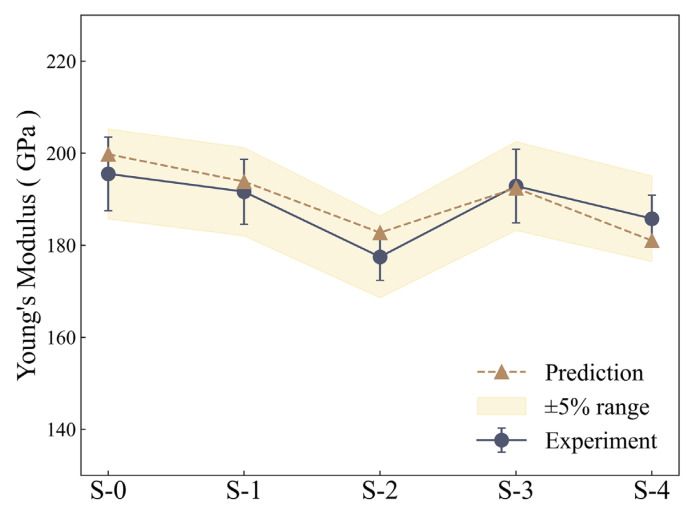
Comparison between the experimental and predicted Young’s modulus of various samples.

Based on the quantitative microstructural data obtained in Section 3.1 and the experimentally measured Young’s modulus, the model constants α and β were optimized by minimizing the sum of squared residuals. The optimized values were α=0.3 and $\beta =1.1\times 10^{6}$. A comparison of model predictions and experimental values is shown in Figure 6, where all predictions fall within the 95% prediction accuracy range (±5%). Additionally, the mean absolute percentage error (MAPE) is 1.59%, which is calculated using the following formula:

(10) $$MAPE=\frac{1}{K}\sum_{i=1}^{K}\left|\frac{P_{i}-E_{i}}{E_{i}}\right|$$

where K is the number of data points and $P_{i}$ and $E_{i}$ represent the model-predicted value and experimentally measured value of the $i_{th}$ sample, respectively.

## 4. Conclusions

In this work, we quantitatively characterized the microstructures—including precipitates and dislocations—in 800H alloy samples at different creep stages, revealing their evolution patterns and gathering detailed data on microstructural changes during creep. The Young’s modulus variation was measured via nanoindentation, and the influence of diverse microstructural factors on the modulus was analyzed. A quantitative predictive model for Young’s modulus based on microstructural features was developed, achieving over 95% accuracy, with a mean absolute percentage error (MAPE) of 1.59% between the predicted and experimental values. This study not only elucidates the intrinsic relationship between microstructures and macroscopic properties but also provides a quantitative reference and theoretical foundation for the future in-service assessment of creep damage through nondestructive testing techniques.

## Data Availability

The original contributions presented in this study are included in this article, and further inquiries can be directed to the corresponding author.
